# Supplementary material for: Infection of Human Endothelial Cells with Lassa Virus Induces Early but Transient Activation and Low Type I IFN Response Compared to the Closely-Related Nonpathogenic Mopeia Virus
Source: Viruses. 2022 Mar 21;14(3):652. doi: 10.3390/v14030652 (PMC8953476; doi:10.3390/v14030652)
Supplement: Supplementary file 1 [file viruses-14-00652-s001.zip › viruses-1634246-supplementary.pdf]

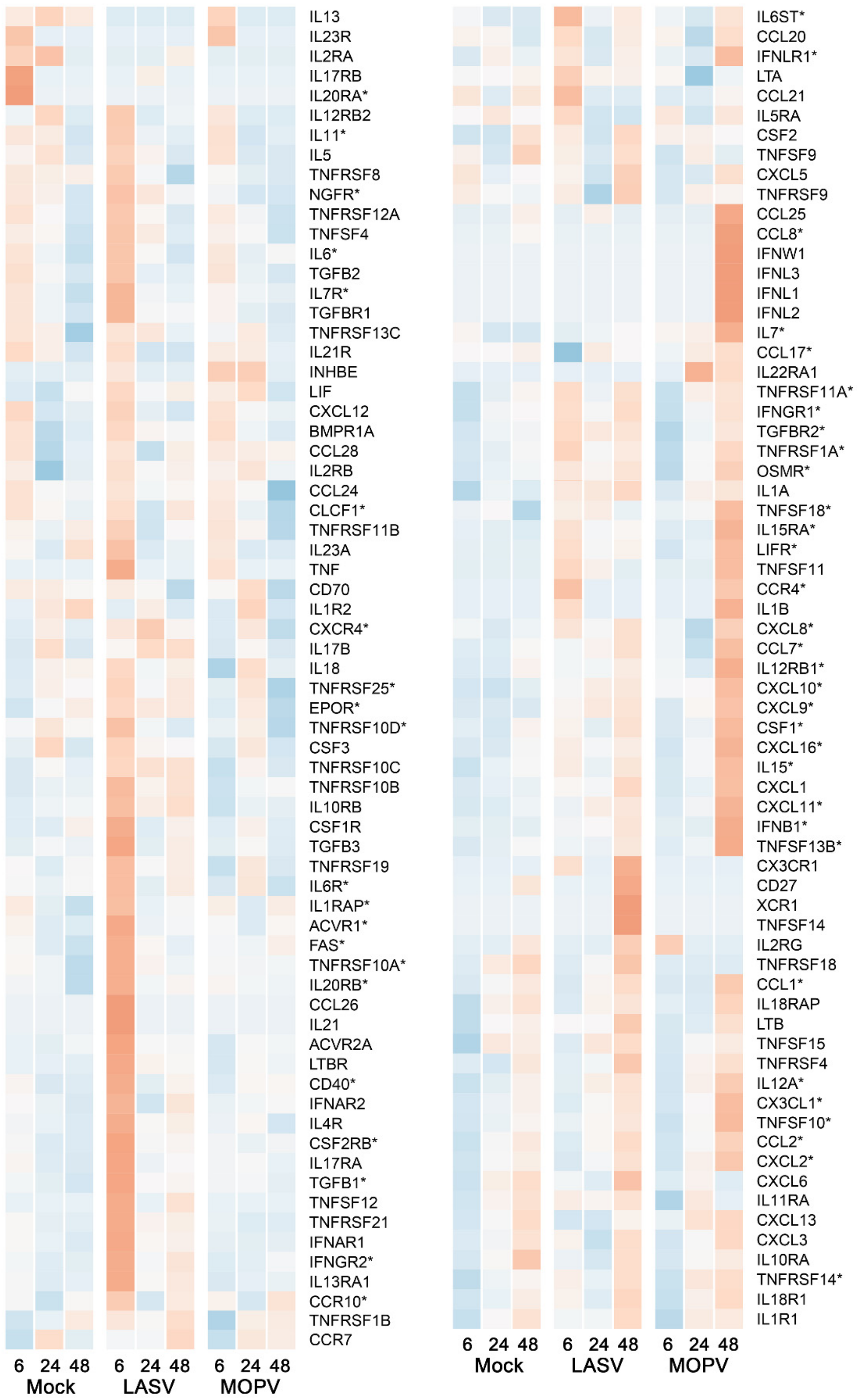

Figure S1. Heatmaps of the gene expression of gene sets related to the cytokine/chemokine response. DE genes are highlighted with asterisks. Gene expression was standardized by VST transformation, centered, and scaled to make the gene expression comparable, hence averaged by condition and timepoint. An enrichment test was performed, using a one-tailed Fisher test. P values were adjusted for multiple comparisons using the Benjamini–Hochberg correction.
